# Partial monosomy 7q34-qter and 21pter-q22.13 due to cryptic unbalanced translocation t(7;21) but not monosomy of the whole chromosome 21: a case report plus review of the literature

**DOI:** 10.1186/1755-8166-1-13

**Published:** 2008-06-19

**Authors:** Svetlana G Vorsanova, Ivan Y Iourov, Victoria Y Voinova-Ulas, Anja Weise, Victor V Monakhov, Alexei D Kolotii, Ilia V Soloviev, Petr V Novikov, Yuri B Yurov, Thomas Liehr

**Affiliations:** 1 Institute of Pediatrics and Children Surgery, Roszdrav, Moscow, Russia; 2 National Research Center of Mental Health, Russian Academy of Medical Sciences, Moscow, Russia; 3 Institute of Human Genetics and Anthropology, Friedrich Schiller University, Jena, Germany

## Abstract

**Background:**

Autosomal monosomies in human are generally suggested to be incompatible with life; however, there is quite a number of cytogenetic reports describing full monosomy of one chromosome 21 in live born children. Here, we report a cytogenetically similar case associated with congenital malformation including mental retardation, motor development delay, craniofacial dysmorphism and skeletal abnormalities.

**Results:**

Initially, a full monosomy of chromosome 21 was suspected as only 45 chromosomes were present. However, molecular cytogenetics revealed a de novo unbalanced translocation with a der(7)t(7;21). It turned out that the translocated part of chromosome 21 produced GTG-banding patterns similar to original ones of chromosome 7. The final karyotype was described as 45,XX,der(7)t(7;21)(q34;q22.13),-21. As a meta analysis revealed that clusters of the olfactory receptor gene family (ORF) are located in these breakpoint regions, an involvement of OFR in the rearrangement formation is discussed here.

**Conclusion:**

The described clinical phenotype is comparable to previously described cases with ring chromosome 21, and a number of cases with del(7)(q34). Thus, at least a certain percentage, if not all full monosomy of chromosome 21 in live-borns are cases of unbalanced translocations involving chromosome 21.

## Background

Non-mosaic monosomy of chromosome 21 is suggested to be incompatible with life as such cases have been occasionally detected in spontaneous abortions [1-3]. To our knowledge there was only one report on full monosomy 21 diagnosed prenatally with a delivery of a male newborn with multiple congenital malformations who has not survived beyond the first day of life [4]. Moreover, the only monosomy potentially viable in humans seems to be that of the X-chromosome [5]. However, in the literature, a number of cytogenetic reports concerning 'full monosomy of chromosome 21' in live-born children can be found. These contradictory findings usually are explained by undetected mosaicism including a normal cell line in different tissues, or are attributed to unbalanced translocations appearing as the loss of chromosome 21 [6].

Here, we describe a case of a female patient with multiple congenital malformations referred to as a non-mosaic monosomy of chromosome 21 after GTG-banding, which, after application of molecular cytogenetic techniques, turned out to be the first case with an unbalanced translocation of chromosomes 7 and 21.

## Results

### Case presentation

The patient, a 2 1/2-years-old girl suffering from mental retardation, motor development delay, craniofacial dysmorphism and skeletal abnormalities, was the first child of non-consanguineous parents, born in 40^th ^week gestation. Both in mother (24 years) and in father (35 years) had no family history of mental retardation or developmental delay. A paternal grandmother has experienced a pregnancy resulted in a male stillbirth at 28 weeks of gestation.

**Figure 1:**
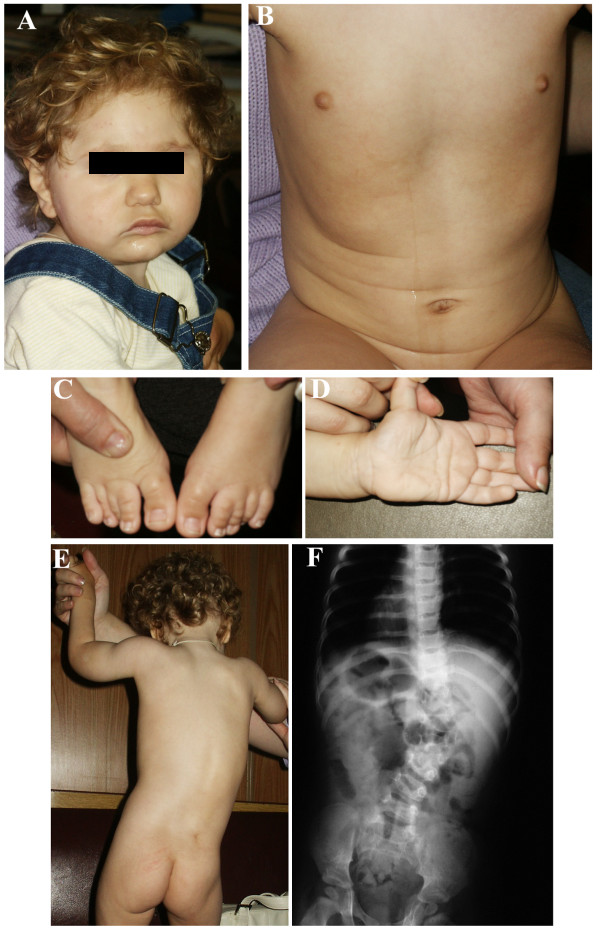
**(A) **Facial appearance of reported patient. **(B) **pectus excavatum. **(C) **partial cutaneous syndactily of the second and third toes. (**D**) transverse palmar crease. **(E) **scoliosis. **(F) **Abnormality of lumbar spine (additional cone-shaped hemivertebra between II and III lumbar vertebrae).

The pregnancy was associated with intrauterine growth retardation. The newborn was hypoplastic with a birth weight of 1830 g (<3. centile), a birth length of 44 cm (<3. centile) and occipitofrontal head circumference (OFC) of 30 cm (<3. centile). At birth, facial dysmorphism, large dysplastic ears, arachnodactyly and congenital scoliosis were noticed. Mental and motor developments were retarded. Specific developmental milestones were delayed: turning did not occur until 13^th ^month and free sitting until 19^th ^months of age. Supported walking started at 2 1/2 years. Speech development was not achieved despite unaffected hearing function as to audiometric investigations. Clinical examination at the age of 2 1/2 years showed length 90 cm (50. centile), weight 11 kg (5 centile). Severe microcephaly with OFC 43 cm (<3. centile) and profound mental retardation were obvious. Furthermore, urinary and intestinal incontinence was revealed. Muscular hypotonia was marked. Craniofacial dysmorphisms manifested as microbrachycephaly, hypotelorism, short and upslanted palpebral fissures, broad nasal tip, micrognathia, large dysmorphic ears, and long philtrum. Curly scalp hair despite straight hair in parents was noticed. She had short neck, arachnodactyly, transverse palmar crease, partial cutaneous syndactily of the second and third toes, pectus excavatum and scoliosis (Figs. 1A, 1B, 1C, 1D, 1E). Ophthalmologic examination revealed hypermetropia of high degree and strabismus convergens alternans. Echocardiography showed mitral valve prolapse. Ultrasonography of kidneys revealed double renal pelvis. X-ray detected abnormality of lumbar spine resulting in lateral curvature of spinal column (Fig. 1F). Atrophy of prefrontal cortex and dilatation of lateral and third ventricles were found on magnetic resonance imaging of the brain (T1 and T2 weighting).

### Cytogenetics

**Figure 2:**
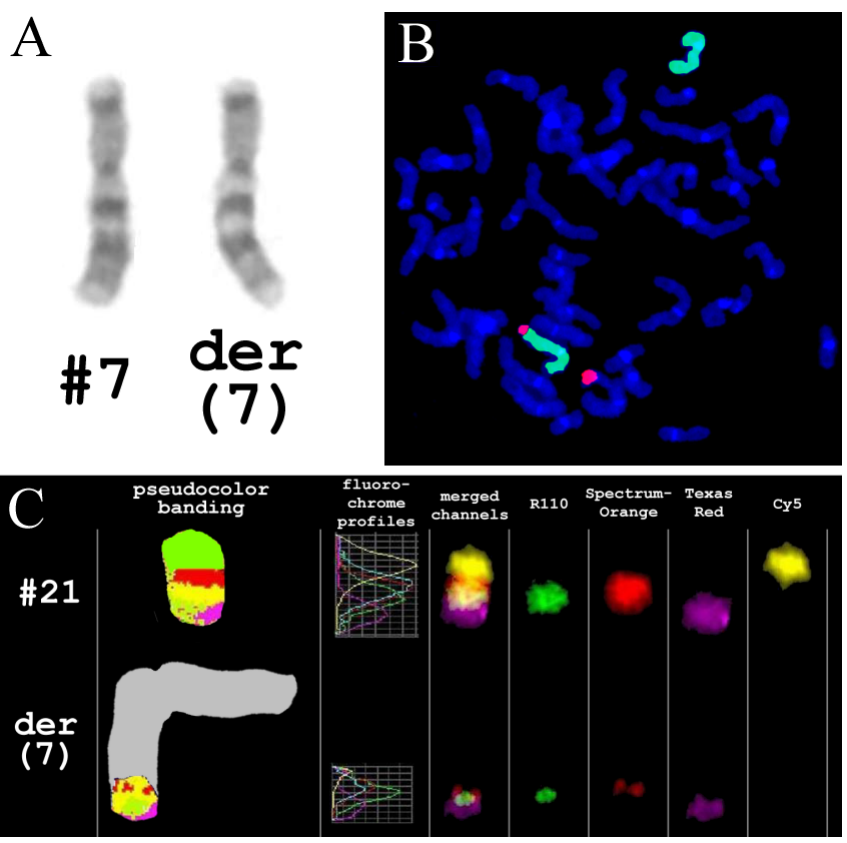
**(A) **GTG-banding appearance of chromosomes 7, note the similarity of banding patterns. **(B) **FISH with whole chromosome painting (WCP) probes for chromosomes 7 (green) and 21 (red) showed a translocation involving these chromosomes. **(C) **Multicolor banding (MCB) analysis of chromosome 21 revealed the translocation to be unbalanced due to the loss of 21pter-q22.13 (R110 signals correspond to q21-q22.2 chromosome 21 region; SpectrumOrange signals – q11.1-q21 chromosome 21 region; TexasRed signals – q21-q22.3 chromosome 21 region; Cyanine 5 signals – p-arm and centromeric region of chromosome 21).

**Figure 3:**
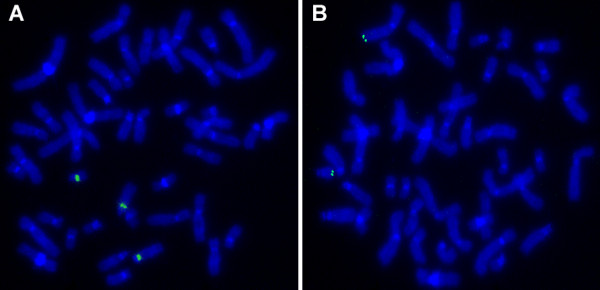
FISH with D13Z1/D21Z1 (**A**) and MCG-P-2C-01 (**B**) probes (for more details see also Table 1).

Cytogenetic analysis revealed an abnormal female karyotype demonstrating the lack of one of homologous chromosome 21 in all the 40 metaphase spreads examined. Even though possible changes of banding patterns within the proximal part of long arm of one of homologous chromosome 7 were assumed (Fig. 2A) the GTG-banding analysis was found not to be sufficient enough to come to a final conclusion. In order to clarify whether the reported case was associated with a translocation involving chromosomes 7 and 21, a series of fluorescence *in situ *hybridization (FISH) experiments were carried out. First, FISH using whole chromosome painting probes (WCP) for chromosomes 7 and 21 were applied. The analysis revealed an imbalanced translocation event involving chromosomes 7 and 21 in all 100 metaphases spreads examined (Fig. 2B). This rearrangement was further characterized by multicolor banding (MCB) for chromosome 21 [7]. The analysis revealed the loss of 21pter-q22.13 due to unbalanced translocation t(7;21) (Fig. 2C). In order to assess the size of the loss within 7q and to define the exact size of the monosomy of 21q, FISH experiments with centromeric and site-specific DNA probes (Table 1; Fig. 3) were performed. Taking into account the data of molecular cytogenetic studies the chromosome abnormality was concluded to be partial monosomy 7q34-qter and 21pter-q22.13 due to an unbalanced translocation t(7;21). Thus, the karyotype was established as 45,XX,der(7)t(7;21)(q34;q22.13),-21. The GTG banded karyotyping and FISH using WCP7 and WCP21 probes showed that the parents had normal karyotypes. Therefore, chromosome abnormality detected was defined as *de novo*.

**Table 1:** Summary of FISH studies using site-specific DNA probes.

DNA probe	Mapped	chromosome 7	t(7;21)	chromosome 21
D7Z1	7cen	+	+	--
6.3.J	7q31	+	+	--
2.6.G	7q34	+	+	--
170.4.E	7q35	+	--	--
PAC 3K23	7q36	+	--	--
D13Z1/D21Z1	13 cen and 21 cen	--	--	+
881D2	21q11.2	--	--	+
MCG-P-320-01	21q22.3	--	+	+
MCG-P-2C-01	21q22.3	--	+	+

DNA probes are derived from the collection of laboratory of cytogenetics of National Research Center of Mental Health RAMS [20-22] except the probe for 7q36 that was kindly provided by Dr. Lyndal Kearney (London, UK).

## Discussion

Despite major developments in cytogenetic techniques made throughout last three decades, routine diagnosis using standard GTG banded karyotyping is still facing cases with unexpected findings [3,6]. A chromosomal abnormality initially diagnosed as a 'full monosomy of chromosome 21' is one of those and the suggested fetal lethality of monosomy 21 is then the indication for further cytogenetic investigations of such cases [6].

One unique description of a comprehensively investigated live born child with presumably non-mosaic monosomy 21 had demonstrated the loss of chromosome 21 to produce exceedingly severe congenital malformations incompatible with life and defined monosomy 21 as an extremely rare chromosome abnormality in live born [4]. Moreover, reviewing the literature indicated that no fewer than 9 cases of unbalanced translocation involving chromosome 21 identified by FISH or molecular genetic studies of initially diagnosed 'full monosomy 21' were reported. Among them, five cases were re-diagnosed as t(5p;21q), two cases as t(11;21)(q24;q22.2), and one case, each, as t(4q;21q), t(18q;21q), and t(X;21), respectively [8-16]. Additionally, a case of low-level mosaic trisomy 21 in an individual with fragile × syndrome was reported [17]. Thus, up to now, no similar cases involving chromosome 7 and 21 were described.

The majority of cases reported were *de novo *unbalanced translocations [8,10,11,14,15], suggesting the formation of such chromosome abnormalities being due to a reciprocal translocation involving chromosome 21 followed by the loss of one of the derivative chromosomes, regardless having an active centromere. As the phenotypic manifestations of these cases are variable, the clinical picture is more likely to be determined by the loss of other chromosome regions except those of chromosome 21.

**Figure 4:**
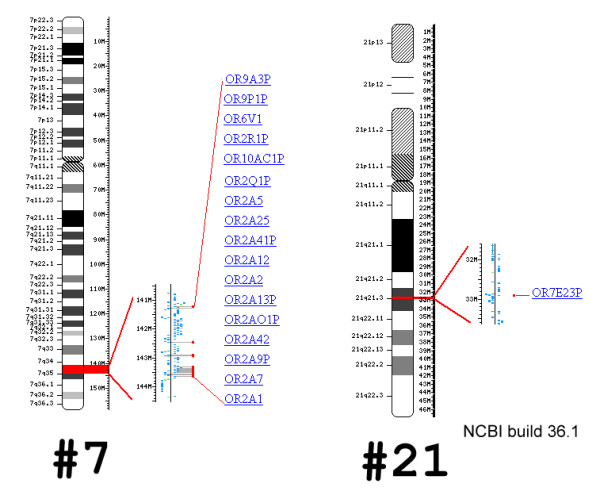
Analysis of breakpoint regions of the index case in the NCBI build 36.1 database depicting the clusters of the olfactory receptor gene family (ORF) to be located in these two chromosomal regions.

Unfortunately, the exact breakpoints were not detailed for almost all of the aforementioned previously reported cases with cryptic translocations involving chromosome 21. In our case, the breakpoints were determined to be in 7q34 and 21q22.13. Interestingly, a check of these breakpoint regions in the NCBI build 36.1 database revealed that clusters of the olfactory receptor gene family (ORF) are located in these two regions (Fig. 4). It is known that these ORF regions can be involved in unequal crossing over and promote translocations between different regions of the genome [18]. Thus, an involvement of OFR in the formation of the rearrangement of at least the reported case and probably in other 'cryptic full monosomy 21 cases' cannot be neglected and should be clarified in further studies.

As the proximal part of chromosome 21 is known to carry less genes than chromosome 7qter, it was reasonable to suggest that main clinical features of the reported case could be similar to those described previously in cases with del(7)(q34) [6]. In accordance with these considerations, actually a number of phenotypic features such as renal abnormalities, microcephaly, atrophy of prefrontal cortex, short neck, 2/3 syndactyly of toes and multiple minor anomalies including epicanthic folds, upstanding palpebral fissures, low-set ears corresponded to previous clinical data on cases with del(7)(q34). Nonetheless, the phenotypic appearance was found also surprisingly similar to a previously described case of ring chromosome 21 [19]. Common features of r(21) and present case were characteristic craniofacial dysmorphism (microbrachycephaly, hypotelorism, short and upslanted palpebral fissures, broad nasal tip, micrognathia, large dysmorphic ears, and long philtrum) and curly scalp hair. Thus, the contribution of 21q loss may be significant for the clinical findings, as well. However, common phenotypic features of chromosome abnormalities as mental retardation, motor development delay and intrauterine growth retardation are most likely to refer to the combined effect of simultaneous loss of both 7q and 21q.

## Conclusion

Since the appearance of G-banded derivate chromosome may be similar to the original GTG banding as it was the case of chromosome 7 in the present case report, molecular cytogenetic techniques represent the most convenient way to prove or refute initial diagnosis. Thus, when analyzing cases that appear to be a 'full monosomy of chromosome 21' or partial monosomy of chromosome 21 due to unbalanced translocations, the application of high resolution molecular cytogenetic techniques (e.g. multi-probe FISH, MCB, or CGH) is unavoidable. Although the latter may appear evident, further cases of unbalanced translocations involving chromosome 21 seems to be required in order to improve subsequent clinical and cytogenetic diagnosis of cases suggested to be a case of monosomy involving the proximal gene-poor region of the chromosome 21 with the precision of breakpoints location.

## Methods

### Cytogenetics

Peripheral blood samples of the patient and her parents were cultivated, harvested and GTG-banded according to standard cytogenetic protocols [20].

### DNA probes

FISH experiments were carried out using whole chromosome painting probes (WCP) for chromosomes 7 and 21 [21] and multicolor banding (MCB) for chromosome 21 [7]. Additionally, two-color-FISH experiments were done using the probes specified in Table 1, which are included in the original collection of laboratory of cytogenetics of National Research Center of Mental Health RAMS [22-24] (for details see also Table 1).

### FISH

FISH was performed according to previously described protocols [21-24]. Multicolor banding (MCB) was generated on methaphase chromosomes as detailed earlier [7].

### Statement

The research done here was carried out in compliance with the Helsinki Declaration – the ethical committee of the National Research Center of Mental Health (RAMS), Moscow approved the study.

## Competing interests

The authors declare that they have no competing interests.

## Authors' contributions

SGV participated in the design of the study, analyzed clinical and cytogenetic data, and drafted basically the manuscript, IY I participated in the design of the study, drafted basically the manuscript and evaluated the FISH-studies, VY V-U picked up the case and was involved in the clinical studies and description, A W performed, evaluated and interpreted the sophisticated molecularcytogenetic studies (MCB-studies), VV M was involved in the molecular cytogenetic studies, AD K performed, evaluated and interpreted the basic evaluated the GTG-banding, IV S contributed DNA probes for site-specific molecular cytogenetic assay, PV N picked up the case and was involved in the clinical studies and description, YB Y participated in the design of the study and drafted the manuscript, T L evaluated and interpreted the sophisticated molecularcytogenetic studies (MCB-studies) and drafted the manuscript. All authors read and approved the final manuscript.

## Consent

A written consent was obtained from the parents of the patient to publish details and pictures of the child.
